# Do Worry and Brooding Predict Health Behaviors? A Daily Diary Investigation

**DOI:** 10.1007/s12529-020-09898-1

**Published:** 2020-05-18

**Authors:** F. Clancy, D. B. O’Connor, A. Prestwich

**Affiliations:** Faculty of Medicine and Health, School of Psychology, The University of Leeds, Leeds, UK

**Keywords:** Worry, Brooding, Rumination, Stress, Health, Behavior

## Abstract

**Background:**

Meta-analyses have reported associations between perseverative cognition (both worry and brooding) and increased engagement in health-risk behaviors, poorer sleep, and poorer physiological health outcomes.

**Method:**

Using a daily diary design, this study investigated the within- and between-person relationships between state and trait perseverative cognition and health behaviors (eating behavior, physical activity, alcohol consumption, and sleep) both cross-sectionally and prospectively. Participants (*n* = 273, 93% students, *M*_age_ = 20.2, SD = 4.11, 93% female) completed morning and evening diaries across 7 consecutive days.

**Results:**

Multilevel modeling analyses revealed that, cross-sectionally, higher levels of state worry were associated with more time spent sitting and higher levels of state brooding predicted less daily walking.

**Conclusion:**

Worry and brooding may represent useful intervention targets for improving inactivity and walking levels, respectively.

**Electronic supplementary material:**

The online version of this article (10.1007/s12529-020-09898-1) contains supplementary material, which is available to authorized users.

## Introduction

It is well established that stress can impact health directly through autonomic and neuroendocrine processes but also, indirectly, by influencing health behaviors. For example, stress has been found to be associated with an increased proinflammatory response [1, 2] which can increase susceptibility to diseases of chronic inflammation, with research supporting links between inflammatory diseases ranging from diabetes [3] to cancer [4]. In terms of the indirect pathway, research supports associations between stress and poorer sleep outcomes [5], greater alcohol consumption [6, 7], unhealthy eating behaviors in adults and children [8–12], and less physical activity [13]. In turn, these behaviors have been linked with poorer health outcomes including increased rates of morbidity and mortality [14–17].

### Perseverative Cognition, Health, and Health Behaviors

Perseverative cognition [18] is defined as the cognitive representation of past stressful events (rumination) or feared future events (worry). The perseverative cognition hypothesis [18] proposes that, in such instances where the physical stressor is absent, the cognitive representation alone can induce the physiological stress response. It is suggested that, when stress is perseverated upon, the damaging physiological activation associated with stress is also protracted, thus increasing susceptibility to stress-related ill-health. Rumination is a key type of perseverative cognition which can broadly be defined as repetitive thinking about negative affect related to stress and its causes, symptoms, and consequences [19]. Rumination can be conceptualized as having both a harmful and an adaptive component: brooding and reflection, respectively [20]. Worry is a related construct, but the focus of the negative, repetitive thoughts is on the future rather than the past [21].

Ten years after the publication of the perseverative cognition hypothesis, Ottaviani et al. [22] conducted a systematic review and meta-analysis of 60 studies investigating the physiological concomitants of perseverative cognition and reported associations between perseverative cognition and higher systolic blood pressure, diastolic blood pressure, heart rate and cortisol, and lower heart rate variability across both experimental and correlational studies. In an extension to the perseverative cognition hypothesis, Clancy et al. [23] proposed that there may be an additional indirect pathway between perseverative cognition and health outcomes via health behaviors. In their meta-analysis, perseverative cognition was associated with more health-risk behaviors. However, many of the included studies were not explicitly designed to investigate links between perseverative cognition and health behaviors. In addition, few studies had employed validated measures of perseverative cognition and there was an over-reliance on cross-sectional measurements.

Due to the small number of published studies (*k* = 19), the Clancy et al. [23] review was unable to isolate associations with particular behaviors, which were instead classified as health-risk or health-promoting. There are a number of behaviors which are particularly important from a public health perspective, and therefore, their association with perseverative cognition requires further investigation. Obesity has been shown to increase the risk of a number of diseases, including coronary heart disease and diabetes [24], and the consumption of high-calorie, low-nutrient foods combined with time spent sedentary contributes to obesity [25]. Furthermore, fast foods, high in fat and sugar, have been associated with increased body weight and poorer metabolic outcomes [14, 15]. In contrast, consumption of fruit and vegetables may have a protective effect on stroke and coronary heart disease risk [26] and some types of cancer [27]. Similarly, physical activity has been found to be widely beneficial to health and health-related quality of life [28]. Finally, alcohol consumption has been shown to increase the risk of cancer and cancer-related death [16]. These behaviors (unhealthy food intake, fruit and vegetable consumption, physical activity, and alcohol intake) are integral to improving population health, and therefore, if perseverative cognition is shown to influence these behaviors, then it may prove to be a valuable target for future behavior change interventions.

In addition, review evidence has been reported for associations between short sleep duration and poorer general, cardiovascular, metabolic, mental, and immunologic health, as well as greater experience of pain and greater overall rates of mortality [17]. A recent meta-analysis found that sleep disturbance was also associated with markers of inflammation [29]. In a subsequent systematic review and meta-analysis of the association between perseverative cognition and sleep outcomes, Clancy et al. [30] reported significant small- to medium-sized associations between both worry and rumination and poorer quality sleep, shorter sleep duration, and longer sleep onset latency. The authors note that the causal direction of the relationship between perseverative cognition and sleep is unknown. This may be important as sleep disturbance has been found to be associated with an increased relative risk of suicidal ideation [31], providing some evidence that poor sleep can lead to negative thought patterns. This led Clancy et al. [30] to suggest that perseverative cognition and sleep may interact in a damaging bidirectional cycle. As bidirectional associations were also not tested in the earlier Clancy et al. [23] meta-analysis, it is of interest to explore the directionality of the associations between perseverative cognition and other health behaviors aside from sleep.

Daily diaries enable the assessment of both state and trait perseverative cognition as analyses can be conducted at both a between-person (trait) and within-person (state) level. Furthermore, such studies allow researchers to capture prospective associations which are closer in time than traditional retrospective recall methods. Additionally, researchers are better able to capture everyday real-world behaviors and participants’ recent states more accurately than global retrospective recall measures due to systematic bias in retrospective recall [32], such as recalling more negative information when in a negative mood [33], or general issues with poor recall. An additional benefit of daily diary methods is that they enable data collection at the level of the individual and at the level of the (sample) population, thus creating a hierarchical structure within the data and enabling multilevel modeling analyses. This type of analysis is valuable in addressing the ecological fallacy, that is, the error that can occur when inferences from population (ecological) level data are applied at the level of the individual [34].

Verkuil et al. [35] found that trait worry only accounted for 24% of the variance in daily worry, and Ottaviani et al. [22] found that state and trait perseverative cognition predicted different physiological outcomes. This suggests that (1) state and trait perseverative cognition may contain elements unique to one another, and (2) state and trait perseverative cognition may predict health behavior outcomes differently. To more fully understand the relationship between perseverative cognition and health behaviors, therefore, it is vital to test the association between both state and trait perseverative cognition and health behaviors. Zoccola et al. [36] found that high stressor-specific (state) rumination predicted longer sleep onset latency and this was higher in participants scoring highly on trait rumination. However, to the authors’ knowledge, this is the only published study which has investigated how state and trait perseverative cognition may interact in predicting health behaviors.

The aim of the current research was to assess the within- and between-person direct and interactive relationships between state and trait perseverative cognition and health behaviors at a daily level, both cross-sectionally and prospectively. An additional aim was to assess whether perseverative cognition is bidirectionally associated with health behaviors at a daily level. As there is little evidence in this area, we did not predict whether the associations between perseverative cognition and concurrent/subsequent health behaviors would be stronger or weaker than the associations between health behaviors and subsequent perseverative cognition. To investigate these aims, participants were asked to complete a survey including demographics and trait measures of perseverative cognition and then complete morning and evening diaries across 7 consecutive days which captured sleep, state perseverative cognition, eating behavior, physical activity, and alcohol consumption.

### Hypotheses

I. Trait perseverative cognition will be positively associated with health-risk behaviors (greater consumption of unhealthy snack foods and alcohol and more time spent sitting) and negatively associated with health-promoting behaviors (lower consumption of fruit and vegetables, lower levels of physical activity, and poorer sleep parameters).
II. Daily perseverative cognition will be positively associated with health-risk behaviors and negatively associated with health-promoting behaviors cross-sectionally (perseverative cognition and health behaviors measured at the same time) and prospectively (health behaviors measured the following day).
III. Positive associations between daily perseverative cognition and health-risk behaviors will be moderated by trait perseverative cognition such that these associations will be stronger as levels of trait perseverative cognition increase.
IV. Negative associations between daily perseverative cognition and health-promoting behaviors will be moderated by trait perseverative cognition such that these associations will be stronger as levels of trait perseverative cognition increase.
V. Daily health behaviors will be associated with daily perseverative cognition prospectively (perseverative cognition measured after health behavior).

## Method

### Design and Participants

A daily diary design was utilized whereby participants completed online diaries twice daily for 7 consecutive days. Evening diaries captured daily stressors, perseverative cognition (worry and brooding), and health behaviors, and morning diaries captured variables relating to the previous night’s sleep. Participants were recruited via an email participant pool used by staff and students at the university and via posters, social media advertisements, and word of mouth. Participants were excluded from participating if they were not fluent in English or were under 18. Ethical approval was granted by the university’s local ethics committee (reference: 16-0291, date of approval: 02 November 2016). A total of 329 participants were recruited to the study and completed a total of 2063 evening diaries and 2117 morning diaries. Of these, 273 completed 4 or more eligible evening diaries and were therefore eligible to be included in data analyses. This equated to 1645 evening diaries and 1095 diaries in which a morning diary could be matched to the previous evening’s diary. Consistent with Gartland et al. [37], a cut-off of 4 diaries was decided prior to analysis to allow sufficient daily comparisons for within-person analyses. Undergraduate psychology students received credits for their participation and were entered into a shopping voucher prize draw. All other participants were only entered into the prize draw. A university ethics committee approved the study. The participants (93% female; 91% British; 93% university students) included in the analyses (those that completed 4 or more diaries) had a mean age of 20.2 years old (SD = 4.11) and a mean body mass index (BMI) of 22.04 (SD = 4.04).

### Measures

#### Trait Perseverative Cognition

*Trait worry* was measured using the 16-item Penn State Worry Questionnaire [38]. Items include ‘my worries overwhelm me’ and ‘many situations make me worry.’ Responses were rated on a 5-point scale (1 = ‘not at all typical of me,’ 5 = ‘very typical of me’). Five items were reverse scored (e.g., ‘I do not tend to worry about things’) so that a higher score represented a higher level of trait worry (*α* = 0.92). This measure has previously been shown to be reliable and valid [39]. *Trait brooding* was measured using 5 items from the validated brooding subscale of the Ruminative Responses Scale [20]. Responses were rated on a 4-point scale (‘almost never,’ ‘sometimes,’ ‘often,’ and ‘almost always’) and items include ‘What am I doing to deserve this?’ and ‘Why do I always react this way?’. Higher scores relate to higher levels of brooding (*α* = 0.78).

#### Morning Diary

Participants were asked to report how long they slept in total (*total sleep time*) and how long it took them to fall asleep (*sleep onset latency*). *Sleep quality* was assessed with one item whereby participants were asked to rate from 1 to 5 (‘not at all’ to ‘very’) how tired they felt that morning. Higher scores refer to longer sleep onset latency, more sleep time, and poorer sleep quality. Single-item measures were used to reduce participant burden. These items were adapted for daily use from the Pittsburgh Sleep Quality Index [40].

#### Evening Diary

Participants were asked to report how often they worried and brooded. Descriptions and examples of worry and brooding were provided for participants. *Daily worry* and *daily brooding* questions were adapted from Zoccola et al. [41]. These were, ‘Today, how often did you worry or focus on negative things that may occur or happen to you in the future?’ and ‘Today, how often did you ruminate or dwell over negative things that happened to you or upset you in the past (including today)?’. ‘How often’ was added to both items to measure the frequency of these thoughts and ‘including today’ was added to the rumination item to emphasize to participants that rumination over that day’s events was relevant. Unlike the yes/no response of Zoccola et al. [41], here, a 5-point scale (‘never’ to ‘very often’) was used to again capture the frequency of these thoughts. Although the items refer to rumination, their focus on negative components of rumination, rather than the reflective components, means the measure is best characterized as brooding [19, 20].

Like O’Connor et al. [11], participants reported the number of between-meal snacks they ate that day. These were coded as high and low fat and sugar by pairs of researchers. If discrepancies could not be resolved through discussion, the lead researcher assisted in the decision-making process. In line with the UK’s National Health Service (NHS) guidelines, *high fat snacks* were categorized as containing 17.5 g of fat or more per 100 g. *High sugar snacks* were categorized as containing 22.5 g or more of sugar per 100 g, as per NHS guidelines. A grocery website was used to obtain this information, and where a brand of food item was not specified, generic items were agreed upon across the group of coders. The total number of high fat and high sugar snacks consumed per day was calculated. Participants were also asked to indicate the portions of fruit and vegetables they had eaten that day. A website link was provided which informed participants of what constitutes as a portion of fruit and vegetables (http://www.nhs.uk/Livewell/5ADAY/Pages/Portionsizes.aspx).

In regard to *alcohol*, participants were asked to report how many pints of beer, cider, or lager they had consumed that day; how many standard sized glasses of wine (175 ml); how many shots of spirits of liqueur (25 ml); and if they had consumed any other alcohol and if so, how much. This information was then converted into alcohol units using the Drinkaware alcohol unit calculator (https://www.drinkaware.co.uk/understand-your-drinking/unit-calculator) by the same pairs of researchers.

*Physical activity* was measured using the International Physical Activity Questionnaire—Short Form [42], which was adapted for daily use by substituting the period of reference from the last 7 days to ‘today.’ Participants were asked to report, in hours and minutes, how long they had spent engaging in vigorous and moderate activity that day and how long they had spent walking. In accordance with the standardized use of the IPAQ-SF, participants were asked to only include this information if they had spent more than 10 min engaging in these activities, and a description of vigorous and moderate activity was provided. Additionally, to assess sedentary behavior, also from the IPAQ-SF, participants were asked to indicate for how long they had spent sitting that day (this was not limited to more than 10 min). As the standard scoring system for the IPAQ-SF is based upon weekly activity, these items were used as single-item measures with the minutes of activity (or sitting) as the outcomes.

### Procedure

Participants attended a session at the university in which they provided informed consent and completed the background survey (including demographics and measures of trait worry and brooding). The following day (or the following Monday if the initial session was on a Friday), participants were emailed a link to the morning diary (6 am) to be completed upon awakening and an evening diary (7 pm) to be completed before bed. These diary emails were sent for 7 consecutive days. After the final day, participants were debriefed via email.

#### Method of Analysis

Data was excluded from analysis if: (1) less than 4 days of diaries were completed, to provide sufficient data for within-person analyses; and (2) morning diaries were completed after 12 pm and if evening diaries were completed after 2 am as backfilled diaries are potentially less accurate [32]. Note that survey links did not expire after the specified time, but the system did register the time that surveys were submitted. Multilevel analyses were conducted using HLM7 software. Daily variables were nested at level 1, and demographic and trait variables at level 2. Level 1 variables were group mean centered and level 2 variables were grand mean centered (apart from sex which was uncentered due to its dichotomy).

For cross-sectional analyses, predictors and outcomes were measured at the same time (in the evening diary) and trait perseverative cognition variables (measured at the initial session) were entered into the models as moderating variables. For prospective analyses, predictors and outcomes were measured on consecutive days. So, either (1) perseverative cognition the previous evening was added as the predictor of sleep outcomes (measured the following morning) or other health behavior outcomes (measured the following evening), with trait perseverative cognition variables added as moderators, or (2) health behaviors were added as predictors of perseverative cognition (measured later the same day in the case of sleep, or measured the following evening day in the case of other health behaviors), without moderating variables as there was no theoretical justification to test cross-level interactions in these analyses. In all prospective analyses, the outcome from the previous day (either perseverative cognition or behavior) was added as a covariate.

These differential analyses necessitated datasets which differed in their configuration. This amounted to five datasets: (1) perseverative cognition from 1 day’s evening diary was matched to health behaviors (apart from sleep) from the same evening diary (this was the only cross-sectional dataset and included no covariates), (2) perseverative cognition from 1 day’s evening diary was matched to sleep outcomes from the following day’s morning diary (covarying for the previous day’s sleep), (3) perseverative cognition from 1 day’s evening diary was matched to health behaviors (apart from sleep) from the following day’s evening diary (covarying for the previous day’s health behavior), (4) sleep outcomes from 1 day’s morning diary were matched to perseverative cognition from the same day’s evening diary (covarying for the previous day’s perseverative cognition), and (5) health behaviors (apart from sleep) from 1 day’s evening diary were matched with perseverative cognition measured in the following day’s evening diary (covarying for the previous day’s perseverative cognition).

Note that due to the large number of analyses, a Bonferroni correction was applied to reduce the type 1 error rate. This consisted of dividing the alpha level by the number of comparisons [43]. Outcomes which would typically be considered significant (*p* < 0.05) were not interpreted as such here unless they met the corrected alpha level. Alphas were corrected per block of analyses, that is, cross-sectional analyses (0.05/18 = *p* ≤ 0.003, see Table 2): prospective analyses where worry and brooding were the predictor variables (0.05/24 = *p* ≤ 0.002, see Table S1 in the Supplementary Material) and prospective analyses where worry and brooding were the outcome variables (0.05/24 = *p* ≤ 0.002, see Table S2 in the Supplementary Material).

In order to account for relevant covariates in the analyses, the associations between age and BMI and all outcome variables were assessed (including worry and brooding). There was too little variation within sex, education, and employment status to assess these associations. Analyses revealed significant associations between age and moderate activity (*β*_01_ = − 0.36, *p* = 0.003), vigorous activity (*β*_01_ = − 0.49, *p* < 0.001), and sitting (*β*_01_ = − 2.90, *p* = 0.002). There were no significant associations with BMI. Age was added as a covariate where associations between perseverative cognition and health behaviors met the corrected significance level.

#### Treatment of Missing Data

The percentage of missing data was analyzed across the final datasets (participants completing 4 or more diaries/matched diaries). Missing value analysis was conducted on the full datasets before totals had been computed. Less than 1% of data was missing overall. Expectation maximization chi-square tests [44] were nonsignificant (*p* > 0.05) for all datasets, confirming that data was missing completely at random. As levels of missing data were minimal and missingness was random, an expectation maximization method was used to impute missing data [45]. As this method is only appropriate for continuous data, this left one data point missing at level 2 (on sex) which was subject to listwise deletion when running analyses.

#### Attrition Analyses

A MANOVA was conducted on continuous variables to compare those who completed 4 or more daily diaries in dataset 1 (completers) to those who completed less than 4 daily diaries (dropouts). The MANOVA was statistically significant, *F*(6, 285) = 2.94, *p* = 0.01. Main effects of completion status were found on worry (completers: *M* = 56.30, SD = 11.65; dropouts: *M* = 51.07, SD = 13.72) but not on age, BMI, or brooding (*p* > 0.05). For categorical variables (sex, nationality, ethnicity, employment status, and education), chi-square analyses were conducted, and significant differences were found on sex, *χ*^2^ (*df* = 1) = 15.57, *p* < 0.001, and educational status, *χ*^2^ (*df* = 4) = 10.75, *p* = 0.03, across dropouts and completers. Dropouts consisted of a higher percentage of male participants and were more highly educated, compared to completers. No significant differences were found across nationality, ethnicity, or employment status (*p* > 0.05).

## Results

### Descriptive Statistics

Descriptive statistics for level 1 (within-person) and level 2 (between-person) variables from datasets 1 and 2 are reported in Table 1. Also see Table 1 for sample size details of datasets 3–5.

**Table 1:** Descriptive statistics for the daily (level 1) and between-person (level 2) measures

	Dataset 1				Dataset 2		
	Mean (SD)	Minimum	Maximum		Mean (SD)	Minimum	Maximum
Trait worry	55.83 (11.80)	26.00	80.00	Trait worry	55.38 (11.57)	27.00	97.00
Trait brooding	10.23 (3.19)	5.00	19.00	Trait brooding	10.16 (3.16)	5.00	19.00
Daily worry	2.82 (1.15)	1.00	5.00	Daily worry	2.90 (1.15)	1.00	5.00
Daily brooding	2.37 (1.19)	1.00	5.00	Daily brooding	2.36 (1.17)	1.00	5.00
High fat snacks	0.82 (0.82)	0.00	4.00	Sleep onset latency^a^	31.11 (34.52)	0.00	174.00
High sugar snacks	0.71 (0.73)	0.00	3.00	Total sleep time	456.55 (99.37)	0.00	740.00
Fruit portions	1.27 (1.24)	0.00	8.00	Sleep quality	3.04 (1.14)	1.00	5.00
Vegetable portions	2.00 (1.40)	0.00	8.00				
Alcohol units	1.16 (3.12)	0.00	37.70				
Vigorous activity^a^	11.73 (30.45)	0.00	184.00				
Moderate activity^a^	11.73 (38.69)	0.00	235.00				
Walking^a^	71.95 (80.82)	0.00	341.00				
Sitting^b^	402.85 (184.82)	31.00	1320.00				

Only datasets 1 and 2 are included for brevity as descriptive statistics were similar across datasets. Dataset 1: level 1 (*n* = 1638), level 2 (*n* = 272). Dataset 2: level 1 (*n* = 809), level 2: (*n* = 160). Dataset 3: level 1 (*n* = 1132), level 2 (*n* = 217). Dataset 4: level 1 (*n* = 923), level 2 (*n* = 181). Dataset 5: level 1 (*n* = 1122), level 2 (*n* = 215)

^a^Due to extreme outliers at the upper end, vigorous physical activity, moderate physical activity, walking, and sleep onset latency were truncated to 2 SDs above the mean

^b^Due to extreme outliers at the lower end, sitting was truncated to 2 SDs below the mean

### Cross-Sectional Associations Between Perseverative Cognition and Health Behaviors

Daily worry was associated with more minutes spent sitting, *β*_10_ = 12.99, *p* = 0.001, and daily brooding was associated with fewer minutes of daily walking, *β*_10_ = − 6.85, *p* < 0.001 (see Table 2). Worry still significantly predicted sitting, when covarying for age (*β*_10_ = 13.21, *p* < 0.001). Neither state worry nor brooding was associated with high fat or high sugar snacking, fruit or vegetable consumption, moderate or vigorous activity, or alcohol intake. Neither trait worry nor trait brooding was significantly associated with any health behavior outcomes. There were no significant cross-level interactions between state/daily and trait perseverative cognition.

**Table 2:** Cross-sectional associations between state and trait perseverative cognition and health behaviors

	Worry			Brooding		
	*β*	SE	*t*	*β*	SE	*t*
*Intercept:* high fat snacks	0.82^***^	0.03	27.26	0.82^***^	0.03	27.20
Level 1 slope: daily PC	0.02	0.02	1.00	0.02	0.02	0.92
Cross-level interaction with trait PC						
Level 2 slope: trait PC	0.00	0.00	1.20	0.00	0.01	0.39
Trait PC × daily PC	0.00^*^	0.00	2.39	0.01	0.01	0.84
*Intercept:* high sugar snacks	0.70^***^	0.03	27.72	0.70^***^	0.03	27.69
Level 1 slope: daily PC	0.02	0.02	1.10	0.02	0.02	0.96
Cross-level interaction with trait PC						
Level 2 slope: trait PC	− 0.00	0.00	− 1.07	0.01	0.01	0.64
Trait PC × daily PC	0.00	0.00	0.94	− 0.00	0.01	− 0.21
*Intercept:* fruit	1.26^***^	0.05	22.94	1.26^***^	0.05	22.94
Level 1 slope: daily PC	− 0.01	0.02	− 0.45	− 0.00	0.03	− 0.06
Cross-level interaction with trait PC						
Level 2 slope: trait PC	0.00	0.00	0.27	− 0.00	0.02	− 0.18
Trait PC × daily PC	− 0.00	0.00	− 0.47	0.00	0.01	0.16
*Intercept:* vegetables	1.99^***^	0.06	34.09	1.99^***^	0.06	34.12
Level 1 slope: daily PC	− 0.02	0.03	− 0.65	0.01	0.03	0.21
Cross-level interaction with trait PC						
Level 2 slope: trait PC	0.00	0.00	0.65	− 0.01	0.02	− 0.76
Trait PC × daily PC	− 0.00	0.00	− 0.43	0.00	0.01	0.48
*Intercept:* vigorous activity	11.86^***^	1.07	11.06	11.84^***^	1.07	11.05
Level 1 slope: daily PC	− 2.19^*^	0.77	− 2.84	− 1.21	0.76	− 1.60
Cross-level interaction with trait PC						
Level 2 slope: trait PC	− 0.14	0.09	− 1.57	0.03	0.29	0.11
Trait PC × daily PC	0.06	0.07	0.83	0.02	0.25	0.06
*Intercept:* moderate activity	11.92^***^	1.20	9.92	11.95^***^	1.20	9.96
Level 1 slope: daily PC	− 0.40	0.96	− 0.42	− 1.17	1.03	− 1.13
Cross-level interaction with trait PC						
Level 2 slope: trait PC	− 0.16	0.13	− 1.21	0.79	0.40	1.95
Trait PC × daily PC	0.04	0.07	0.65	0.19	0.32	0.59
*Intercept:* walking	71.69^***^	3.01	23.85	71.70^***^	3.00	23.87
Level 1 slope: daily PC—walking	− 2.85	2.01	− 1.42	− 6.85^***^	1.61	− 4.26
Cross-level interaction with trait PC						
Level 2 slope: trait PC	− 0.10	0.30	− 0.35	0.51	0.97	0.52
Trait PC × daily PC	− 0.12	0.19	− 0.66	− 0.56	0.46	− 1.22
*Intercept:* sitting	401.85^***^	8.78	45.78	401.84^***^	8.78	45.77
Level 1 slope: daily PC	12.99^**^	3.93	3.31	10.76^*^	4.24	2.54
Cross-level interaction with trait PC						
Level 2 slope: trait PC	− 0.31	0.77	− 0.41	0.90	2.50	0.36
Trait PC × daily PC	0.49	0.35	1.43	1.98	1.28	1.55
*Intercept:* alcohol	1.18^***^	0.10	12.14	1.18^***^	0.10	12.16
Level 1 slope: daily PC	− 0.13	0.07	− 1.83	0.06	0.09	0.64
Cross-level interaction with trait PC						
Level 2 slope: trait PC	0.00	0.01	0.17	− 0.02	0.03	− 0.78
Trait PC × daily PC	0.00	0.01	0.85	0.02	0.03	0.71

*PC* perseverative cognition

^*^ Significant at the 0.05 level, ^**^ significant at the 0.01 level, ^***^ significant at the 0.001 level

### Prospective Associations Between Perseverative Cognition and Health Behaviors

Neither state worry nor brooding prospectively predicted any health behavior outcomes. There were no significant cross-level interactions between state/daily and trait perseverative cognition on prospective health behavior outcomes. See Table S1 in the Supplementary Material for full details.

### Prospective Associations Between Health Behaviors and Perseverative Cognition

No health behaviors or sleep significantly predicted daily worry or brooding prospectively. See Table S2 in the Supplementary Material for full details.

## Discussion

The study findings indicate that components of state perseverative cognition are associated with less walking and more time spent sitting. Trait perseverative cognition did not predict more health-risk or less health-promoting behaviors, and therefore, hypothesis I was not supported. Daily worry was associated with more time spent sitting and daily brooding was associated with less daily walking, and therefore, hypothesis II, that state perseverative cognition would be associated with more health-risk and less health-promoting behaviors, received limited support. Hypotheses III and IV, that associations between daily perseverative cognition and more health-risk and less health-promoting behaviors would be stronger as levels of trait perseverative cognition increased, were not supported. There was no support for hypothesis V, that daily health behaviors would be associated with daily perseverative cognition prospectively. These findings suggest that, out of a number of health behaviors, only components of physical activity appear to be associated with perseverative cognition and only when perseverative cognition is measured at a state level.

Contrary to the systematic review and meta-analysis by Clancy et al. [30], neither trait nor state worry or brooding predicted sleep onset latency, total sleep time, or sleep quality. However, the current study is notable for testing how perseverative cognition measured the previous day relates to sleep measured the following morning, which overcomes the problem whereby measuring the two at the same time may lead to a mood state bias such that negative affect may lead to negatively biased estimates of other outcomes [32]. Furthermore, this study goes beyond the Clancy et al. [30] meta-analysis by assessing bidirectional associations between perseverative cognition and sleep. When considering the lack of significant findings relating to sleep, it is worth noting that this study employed single-item measures of sleep outcomes to reduce participant burden, which may have failed to accurately capture sleep parameters.

Although Cropley et al. [46] reported an association between work-related affective rumination and unhealthy eating, the current study did not demonstrate an association between perseverative cognition and eating behavior. There were also no associations found with fruit or vegetable consumption which reflects similar findings by Cropley et al. [47] in which no association was found between rumination and healthy foods, including fruits and vegetables. On the other hand, Ferrer et al. [48] found that health worry predicted higher fruit and vegetable intake, although health worry (instigated through threatening communication, for example) may have a greater potential to motivate health-protective behaviors than general worry [49]. As there are only two published studies in this area, future research should investigate the association between perseverative cognition and eating behaviors, perhaps employing more content valid measures of eating behavior.

There was also no association between any trait or state perseverative cognition predictors and daily drinking. In a diary study of college students’ drinking habits, Aldridge-Gerry et al. [53] found that emotional rumination predicted more daily drinking, but including the current study, to date, there are only two daily diary studies which have investigated these relationships, and only seven published studies in total, some of which include adolescent samples. Therefore, more research is needed in this area to better understand the relationship between perseverative cognition and drinking behavior, perhaps employing more diverse and less student-focused samples.

Greater daily worry was associated with more time spent sitting, and greater daily brooding was significantly associated with less daily walking cross-sectionally. These findings are consistent with one study that found an association between health worry and reduced physical activity [50], although other research has reported associations between cancer worry [51] and health worry [52] and more physical activity. However, these studies measured trait perseverative cognition and the current findings suggest that state, rather than trait, perseverative cognition is associated with a lower level of physical activity, and this appears to be true of both worry and brooding. These findings suggest that higher levels of daily perseverative cognition may reduce engagement in physical activity.

Contrary to the hypotheses, trait worry and brooding were unrelated to health behaviors and, unlike Zoccola et al. [36], neither moderated the association between state worry and brooding and health behaviors. The findings of this study therefore suggest that state perseverative cognition is associated with health behaviors (in this case, physical activity) and should be targeted in intervention studies. This may be promising from an intervention perspective, as it is possible that the frequency of daily perseverative cognition may be more susceptible to brief psychological interventions than the trait tendency to engage in perseverative cognition, although these factors are likely intertwined and it would be interesting to assess whether both improve simultaneously as a result of targeted interventions.

The association between perseverative cognition and decreased engagement in physical activity has potential implications for behavioral medicine. This includes the development of intervention studies aimed at reducing levels of perseverative cognition, with the goal of promoting physical activity. Existing literature points to some potentially effective interventions including mindfulness-based and cognitive behavioral therapies [54]. Systematic review evidence suggests that, in patient samples, mindfulness-based stress reduction techniques are associated with better sleep by reducing worry [55] and it is possible that these effects may extend to other behaviors, including exercise. Likewise, a brief postpone worry intervention has been shown to significantly reduce daily worry [56, 57], writing about life goals has been shown to reduce ruminative thinking [58], and a self-compassion intervention has been found to improve sleep quality via reduced rumination [59]. It is recommended that future research tests the effectiveness of such interventions within the context of physical activity promotion.

However, given that associations between perseverative cognition and health behaviors reported in meta-analyses only appear to be small, excluding some associations with sleep [23, 30], it may be more effective to develop perseverative cognition interventions that complement or form part of existing intervention packages designed to modify behavior via changes in other determinants of behavior. Furthermore, health behavior interventions are typically based on theories and models that do not consider perseverative cognition [60]. Thus, future research ought to consider the incremental value of variables such as perseverative cognition over established predictors of health behaviors such as intentions and perceived behavioral control [61], and if there is evidence that perseverative cognition explains additional variance in health behaviors, techniques targeting perseverative cognition may supplement existing interventions.

There were a number of limitations of the current research. First, despite employing daily diary methodology, this study may still have been subject to retrospective recall bias, as outcomes were only measured at two time points in the day and may have been influenced by participant’s emotional state when completing the diary. The outcomes may also simply have been biased due to memory limitations. A better way to utilize daily diary methodology is to measure thoughts, mood states, and behaviors at multiple time points throughout the day, such as in a study by Takano et al. [62] in which repetitive thought and mood were measured at semi-random intervals throughout the day. These methods are most reliable when portable electronic devices are provided. However, such methods can be expensive and burdensome and limit the number of participants that can be recruited at one time, and were therefore not feasible in the current study.

Second, Brosschot and van der Doef [56] found that the duration but not the frequency of worry was predictive of somatic symptoms. Here, only the frequency of daily worry and brooding was captured as it was thought that it would be too difficult for participants to recall the number of minutes each day they were worrying and brooding. A measure of duration is more feasible when multiple daily measurements are taken. In the study by Brosschot and van der Doef [56], a daily pen and paper tally was made of worry episodes which may have made estimating duration at the end of the day easier. In future studies, it is suggested that multiple daily measurements of perseverative cognition and health behaviors are taken and that this includes assessment of the duration of daily perseverative cognition.

Third, this study may have been amiss in not measuring and covarying levels of depression and anxiety. Rumination has been shown to be associated with depression [63], and worry is reported to be a central aspect of anxiety disorders and particularly generalized anxiety disorder [64]. Furthermore, depression and anxiety have been negatively associated with health behaviors such as sleep [65]. As such, depression and anxiety could contribute to explaining the relationships between perseverative cognition and health behaviors and/or be determinants or subsequent outcomes of these relations. Further research should be conducted to identify the role of depression and anxiety in perseverative cognition and health behavior relations.

Future research may also wish to explore the measurement of perseverative cognition and how this relates to health behavior outcomes. Ehring et al. [66] argue that measures of worry and rumination are overly specific and that a broader construct is required to encapsulate perseverative cognition (or repetitive negative thinking). They developed the Perseverative Thinking Questionnaire which focuses upon the repetitiveness of thoughts and the difficulty in disengaging from them. In this vein, it is suggested that future research incorporates a more holistic measure of perseverative cognition as a comparison to specific measures of worry and brooding, both at a state and trait level. Additionally, it is suggested that future research explores how the content of perseverative cognition may relate to health behavior outcomes. For instance, it is speculated that worries about health may have the potential to motivate health-promoting behaviors or reduce engagement in health-risk behaviors. Finally, this study recruited a relatively healthy, young sample, the majority of whom were students. It is possible that this particular group may differ from the general population in their behaviors and/or may have a relatively low exposure to acute and/or chronic stressors. It would be useful to explore these associations further in older, more representative and diverse populations.

In conclusion, the findings from this study provide evidence of cross-sectional associations between state, but not trait, worry and brooding, and less physical activity. Furthermore, this research provides novel evidence of differential associations between types of perseverative cognition and health behaviors. This study has gone beyond the existing literature by assessing the association between types of perseverative cognition (worry and brooding, state and trait) and multiple health behaviors cross-sectionally and prospectively, allowing for targeting of behaviors sensitive to perseverative cognition. Additionally, this study has investigated whether health behaviors are also predictive of perseverative cognition. Findings from this research suggest that perseverative cognition may prove to be a useful target for physical activity intervention studies.

## Electronic Supplementary Material

ESM 1 (DOCX 24 kb)

ESM 2 (DOCX 18 kb)
