# Supplementary material for: Do Worry and Brooding Predict Health Behaviors? A Daily Diary Investigation
Source: Int J Behav Med. 2020 May 18;27(5):591–601. doi: 10.1007/s12529-020-09898-1 (PMC7497422; doi:10.1007/s12529-020-09898-1)
Supplement: Supplementary file 1 — (DOCX 24 kb) [file 12529_2020_9898_MOESM1_ESM.docx]

| Table S1. Prospective Associations between Trait and State Perseverative Cognition and Health Behaviors | | | | | | |
| --- | --- | --- | --- | --- | --- | --- |
|  | Worry | | | Brooding | | |
|  | β | *SE* | t | β | *SE* | t |
| *Intercept:* Following Day’s High Fat Snacks | 0.80^***^ | 0.04 | 21.79 | 0.80^***^ | 0.04 | 21.77 |
| Level 1 Slope: Daily PC | 0.03 | 0.03 | 1.30 | 0.00 | 0.03 | 0.01 |
| Cross-Level Interaction with Trait PC |  |  |  |  |  |  |
| Level 2 Slope: Trait PC | 0.00 | 0.00 | 0.79 | 0.00 | 0.01 | 0.32 |
| Trait PC x Daily PC | -0.00 | 0.00 | -0.12 | 0.01 | 0.01 | 1.16 |
| *Intercept:* Following Day’s High Sugar Snacks | 0.70^***^ | 0.03 | 24.72 | 0.70^***^ | 0.03 | 24.70 |
| Level 1 Slope: Daily PC | 0.04 | 0.02 | 1.86 | -0.01 | 0.02 | -0.59 |
| Cross-Level Interaction with Trait PC |  |  |  |  |  |  |
| Level 2 Slope: Trait PC | -0.00 | 0.00 | -1.13 | 0.01 | 0.01 | 0.98 |
| Trait PC x Daily PC | -0.00 | 0.00 | -0.61 | -0.00 | 0.01 | -0.41 |
| *Intercept:* Following Day’s Fruit | 1.26^***^ | 0.07 | 19.06 | 1.26^***^ | 0.07 | 19.06 |
| Level 1 Slope: Daily PC | 0.04 | 0.03 | 1.21 | 0.04 | 0.03 | 1.13 |
| Cross-Level Interaction with Trait PC |  |  |  |  |  |  |
| Level 2 Slope: Trait PC | 0.00 | 0.00 | 0.51 | 0.01 | 0.02 | 0.41 |
| Trait PC x Daily PC | -0.00 | 0.00 | -0.26 | 0.00 | 0.01 | 0.10 |
| *Intercept:* Following Day’s Vegetables | 2.00^***^ | 0.07 | 29.13 | 2.00^***^ | 0.07 | 29.08 |
| Level 1 Slope: Daily PC | 0.03 | 0.04 | 0.77 | -0.01 | 0.04 | -0.34 |
| Cross-Level Interaction with Trait PC |  |  |  |  |  |  |
| Level 2 Slope: Trait PC | 0.01 | 0.01 | 0.95 | 0.01 | 0.02 | 0.35 |
| Trait PC x Daily PC | -0.00 | 0.00 | -0.45 | -0.00 | 0.01 | -0.29 |
| *Intercept:* Following Day’s Vigorous Activity | 11.26^***^ | 1.17 | 9.66 | 11.27^***^ | 1.17 | 9.63 |
| Level 1 Slope: Daily PC | -0.59 | 0.78 | -0.75 | 0.02 | 0.86 | 0.03 |
| Cross-Level Interaction with Trait PC |  |  |  |  |  |  |
| Level 2 Slope: Trait PC | -0.11 | 0.11 | -1.04 | -0.13 | 0.33 | -0.39 |
| Trait PC x Daily PC | 0.12 | 0.09 | 1.29 | -0.15 | 0.24 | -0.64 |
| *Intercept:* Following Day’s Moderate Activity | 8.73^***^ | 1.02 | 8.55 | 8.77^***^ | 1.02 | 8.61 |
| Level 1 Slope: Daily PC | 0.11 | 0.84 | 0.14 | -1.20 | 0.94 | -1.28 |
| Cross-Level Interaction with Trait PC |  |  |  |  |  |  |
| Level 2 Slope: Trait PC | -0.03 | 0.08 | -0.38 | 0.40 | 0.32 | 1.22 |
| Trait PC x Daily PC | -0.03 | 0.10 | -0.27 | -0.49 | 0.29 | -1.69 |
| *Intercept:* Following Day’s Walking | 75.36^***^ | 3.69 | 20.43 | 75.33^***^ | 3.69 | 20.42 |
| Level 1 Slope: Daily PC – Walking | -2.87 | 2.33 | -1.23 | -0.35 | 2.36 | -0.15 |
| Cross-Level Interaction with Trait PC |  |  |  |  |  |  |
| Level 2 Slope: Trait PC | 0.20 | 0.34 | 0.61 | 0.93 | 1.25 | 0.75 |
| Trait PC x Daily PC | 0.05 | 0.20 | 0.27 | 1.39 | 0.70 | 1.97 |
| *Intercept:* Following Day’s Sitting | 406.59^***^ | 10.08 | 40.34 | 406.60^***^ | 10.10 | 40.27 |
| Level 1 Slope: Daily PC | 10.94^*^ | 4.72 | 2.32 | 5.81 | 5.31 | 1.10 |
| Cross-Level Interaction with Trait PC |  |  |  |  |  |  |
| Level 2 Slope: Trait PC | -0.86 | 0.88 | -0.98 | -1.30 | 2.69 | -0.48 |
| Trait PC x Daily PC | -0.55 | 0.39 | -1.41 | -0.40 | 1.47 | -0.27 |
| *Intercept:* Following Day’s Alcohol | 0.99^***^ | 0.10 | 9.62 | 0.99^***^ | 0.10 | 9.66 |
| Level 1 Slope: Daily PC | -0.14 | 0.12 | -1.22 | -0.04 | 0.11 | -0.32 |
| Cross-Level Interaction with Trait PC |  |  |  |  |  |  |
| Level 2 Slope: Trait PC | 0.00 | 0.01 | 0.30 | -0.05 | 0.03 | -1.45 |
| Trait PC x Daily PC | 0.01 | 0.01 | 1.21 | 0.04 | 0.04 | 1.03 |
| *Intercept:* Following Day’s SOL | 31.26^***^ | 1.67 | 18.68 | 31.20^***^ | 1.68 | 18.56 |
| Level 1 Slope: Daily PC | 2.82^*^ | 1.25 | 2.26 | 3.73 | 1.33 | 2.81^**^ |
| Cross-Level Interaction with Trait PC |  |  |  |  |  |  |
| Level 2 Slope: Trait PC | 0.29^*^ | 0.15 | 2.02 | 0.79 | 0.62 | 1.27 |
| Trait PC x Daily PC | 0.22 | 0.11 | 1.98 | -0.40 | 0.37 | -1.08 |
| *Intercept:* Following Day’s TST | 456.67^***^ | 4.09 | 111.59 | 456.73^***^ | 4.08 | 111.98 |
| Level 1 Slope: Daily PC | -5.49 | 3.93 | -1.40 | -0.25 | 4.51 | -0.06 |
| Cross-Level Interaction with Trait PC |  |  |  |  |  |  |
| Level 2 Slope: Trait PC | -0.44 | 0.34 | -1.28 | -1.87 | 1.13 | -1.65 |
| Trait PC x Daily PC | -0.45 | 0.34 | -1.32 | -2.67^*^ | 1.20 | -2.23 |
| *Intercept:* Following Day’s Sleep Quality | 3.03^***^ | 0.05 | 56.06 | 3.03^***^ | 0.05 | 56.47 |
| Level 1 Slope: Daily PC | -0.00 | 0.04 | -0.09 | -0.01 | 0.05 | -0.26 |
| Cross-Level Interaction with Trait PC |  |  |  |  |  |  |
| Level 2 Slope: Trait PC | 0.01 | 0.00 | 1.72 | 0.04 | 0.02 | 2.37^*^ |
| Trait PC x Daily PC | 0.00 | 0.00 | 1.36 | 0.01 | 0.02 | 0.70 |
| Note. All analyses are adjusted for the previous day’s health behavior; ^*^significant at the .05 level, ^**^significant at the .01 level, ^***^significant at the .001 level. PC = perseverative cognition; SOL= sleep onset latency; TST = total sleep time. | | | | | | |
