# Supplementary material for: Do Worry and Brooding Predict Health Behaviors? A Daily Diary Investigation
Source: Int J Behav Med. 2020 May 18;27(5):591–601. doi: 10.1007/s12529-020-09898-1 (PMC7497422; doi:10.1007/s12529-020-09898-1)
Supplement: Supplementary file 2 — (DOCX 18 kb) [file 12529_2020_9898_MOESM2_ESM.docx]

| *Table S2. Prospective Associations between Daily Health Behaviors and State Perseverative Cognition* | | | | | | |
| --- | --- | --- | --- | --- | --- | --- |
|  | Outcome | | | | | |
|  | Daily Worry | | | Daily Brooding | | |
|  | β | *SE* | t | β | *SE* | t |
| *Intercept:* Following Day’s Perseverative Cognition | 2.83^***^ | 0.05 | 52.85 | 2.35^***^ | 0.06 | 40.83 |
| Level 1 Slope: High Fat Snacks | -0.01 | 0.04 | -0.15 | 0.03 | 0.04 | 0.69 |
| *Intercept:* Following Day’s Perseverative Cognition | 2.83^***^ | 0.05 | 52.85 | 2.35^***^ | 0.06 | 40.83 |
| Level 1 Slope: Higher Sugar Snacks | 0.01 | 0.05 | 0.22 | -0.00 | 0.05 | -0.04 |
| *Intercept:* Following Day’s Perseverative Cognition | 2.83^***^ | 0.05 | 52.85 | 2.35^***^ | 0.06 | 40.831 |
| Level 1 Slope: Fruit | -0.00 | 0.04 | -0.08 | -0.01 | 0.04 | -0.28 |
| *Intercept:* Following Day’s Perseverative Cognition | 2.83^***^ | 0.05 | 52.85 | 2.35^***^ | 0.06 | 40.83 |
| Level 1 Slope: Vegetables | 0.03 | 0.03 | 1.14 | -0.05 | 0.03 | -1.68 |
| *Intercept:* Following Day’s Perseverative Cognition | 2.83^***^ | 0.05 | 52.85 | 2.35^***^ | 0.06 | 40.83 |
| Level 1 Slope: Vigorous Activity | 0.00 | 0.00 | 1.55 | 0.00 | 0.00 | 1.13 |
| *Intercept:* Following Day’s Perseverative Cognition | 2.83^***^ | 0.05 | 52.85 | 2.35^***^ | 0.06 | 40.83 |
| Level 1 Slope: Moderate Activity | -0.00 | 0.00 | -0.49 | 0.00 | 0.00 | 0.33 |
| *Intercept:* Following Day’s Perseverative Cognition | 2.83^***^ | 0.05 | 52.85 | 2.35^***^ | 0.06 | 40.83 |
| Level 1 Slope: Walking | -0.00 | 0.00 | -0.86 | 0.00 | 0.00 | 1.56 |
| *Intercept:* Following Day’s Perseverative Cognition | 2.83^***^ | 0.05 | 52.85 | 2.35^***^ | 0.06 | 40.83 |
| Level 1 Slope: Sitting | 0.00 | 0.00 | 1.04 | -0.00 | 0.00 | -0.49 |
| *Intercept:* Following Day’s Perseverative Cognition | 2.83^***^ | 0.05 | 52.85 | 2.35^***^ | 0.06 | 40.83 |
| Level 1 Slope: Alcohol | -0.01 | 0.01 | -0.40 | 0.02 | 0.01 | 1.62 |
| *Intercept:* Following Day’s Perseverative Cognition | 2.86^***^ | 0.06 | 47.55 | 2.35^***^ | 0.06 | 36.65 |
| Level 1 Slope: SOL | 0.00 | 0.00 | 1.80 | 0.00 | 0.00 | 0.80 |
| *Intercept:* Following Day’s Perseverative Cognition | 2.86^***^ | 0.06 | 47.55 | 2.35^***^ | 0.06 | 36.65 |
| Level 1 Slope: TST | -0.00 | 0.00 | -1.12 | -0.00 | 0.00 | -0.68 |
| *Intercept:* Following Day’s Perseverative Cognition | 2.86^***^ | 0.06 | 47.55 | 2.35^***^ | 0.06 | 36.65 |
| Level 1 Slope: Sleep Quality | 0.09^*^ | 0.04 | 2.55 | 0.04 | 0.04 | 1.02 |
| Note. All analyses are adjusted for the previous day’s PC; ^*^significant at the .05 level, ^**^significant at the .01 level, ^***^significant at the .001 level. PC = perseverative cognition; SOL= sleep onset latency; TST = total sleep time. | | | | | | |
